# Supplementary material for: Effect of Soil Type and In Vitro Proliferation Conditions on Acclimation and Growth of Willow Shoots Micropropagated in Continuous Immersion Bioreactors
Source: Plants (Basel). 2022 Dec 27;12(1):132. doi: 10.3390/plants12010132 (PMC9823317; doi:10.3390/plants12010132)
Supplement: Supplementary file 1 [file plants-12-00132-s001.zip › plants-2104908-supplementary.pdf]

Table S1. ANOVA corresponding to Figure 2, regarding the effect of support material on proliferation rates of willow shoots grown in continuous immersion bioreactors. Shoots were grown in MS medium with half strength nitrates, 0.22 µM BA and 3% sucrose for 8 weeks. Values are the mean ± standard error from 3 replicates with 20 explants each. NS number of shoots, SL length of the longest shoot, MC multiplication coefficient.

	**Source of Variation**	**Sum of squares**	**df**	**Mean square**	**F**	**Significance**
**NS**	Between groups	0.011	1	0.011	0.006	0.940
	Within groups	112.972	58	1.948		
	Total	112.983	59			
**SL**	Between groups	570.025	1	570.025	1.043	0.311
	Within groups	31706.708	58	546.667		
	Total	32276.733	59			
**MC**	Between groups	124.844	1	124.844	11.383	0.001
	Within groups	636.139	58	10.968		
	Total	760.983	59			

Table S2. Two-way ANOVA corresponding to Figure 3, regarding the effect of support material (rockwool and plastic boxes) and sucrose supplementation (0 and 3% sucrose) on proliferation rates of willow shoots grown in continuous immersion bioreactors. Shoots were grown in MS medium with half strength nitrates and 0.22 µM BA for 8 weeks. Values are the mean ± standard error from 3 replicates with 20 explants each. Due to the significant interaction found in MC, Bonferroni's adjustment was applied to detect simple main effects. NS number of shoots, SL length of the longest shoot, MC multiplication coefficient.

	Source of Variation	Sum of squares	df	Mean square	F	Significance
**NS**	Sucrose	4.422	1	4.422	1.646	0.202
	Support	13.135	1	13.135	4.889	0.029
	Sucrose x Support	0.152	1	0.152	0.057	0.812
**SL**	Sucrose	1414.238	1	1414.238	1.546	0.216
	Support	67623.581	1	67623.581	73.947	0.000
	Sucrose x Support	411.138	1	411.138	0.450	0.504
**MC**	Sucrose	738.871	1	738.871	20.231	0.000
	Support	2219.382	1	2219.382	60.770	0.000
	Sucrose x Support	331.491	1	331.491	9.077	0.003

Pairwise comparisons for Multiplication Coefficient after Bonferroni adjustment

Support x Sucrose

Support	I (Sucrose)	J (Sucrose)	Mean Difference (I-J)	Std. error	Significance
**Cubes**	0%	3%	1.553	1.406	0.271
	3%	0%	-1.553	1.406	0.271
**Boxes**	0%	3%	7.852*	1.548	0.000
	3%	0%	-7.852*	1.548	0.000

Sucrose x Support

Sucrose	I (Support)	J (Support)	Mean Difference (I-J)	Std. error	Significance
**0%**	Cubes	Boxes	-11.299*	1.521	0.000
	Boxes	Cubes	11.299*	1.521	0.000
**3%**	Cubes	Boxes	-5.000*	1.435	0.001
	Boxes	Cubes	5.000*	1.435	0.001

Table S3. Two-way ANOVA corresponding to Figure 4, regarding the effect of support material (rockwool cubes and plastic boxes) and sucrose supplementation (0 and 3% sucrose) on pigment content of willow shoots grown in continuous immersion bioreactors. Shoots were grown in MS medium with half strength nitrates and 0.22 µM BA for 8 weeks. Due to the significant interaction found in all the parameters Bonferroni's adjustment was applied to detect simple main effects.

	**Source of Variation**	**Sum of squares**	**df**	**Mean square**	**F**	**Significance**
**Chlorophyll a**	Sucrose	282503.799	1	282503.799	25.190	0.000
	Support	398.931	1	398.931	0.036	0.851
	Sucrose x Support	118897.232	1	118897.232	10.602	0.002
**Chlorophyll b**	Sucrose	141411.692	1	141411.692	26.708	0.000
	Support	3137.888	1	3137.888	0.593	0.446
	Sucrose x Support	43428.026	1	43428.026	8.202	0.006
**Carotenoids**	Sucrose	15010.193	1	15010.193	21.628	0.000
	Support	79.892	1	79.892	0.115	0.736
	Sucrose x Support	10019.571	1	10019.571	14.437	0.000
**Pairwise comparisons after Bonferroni adjustment for variables with interaction p value ≤ 0.05**						
**Support x Sucrose**						
**Dependent variable**	**Support**	**I (Sucrose)**	**J (Sucrose)**	**Mean Difference (I-J)**	**Std. error**	**Significance**
**Chlorophyll a**	Cubes	0%	3%	55.494	41.478	0.188
		3%	0%	-55.494	41.478	0.188
	Boxes	0%	3%	260.480*	47.360	0.000
		3%	0%	-260.480*	47.360	0.000
**Chlorophyll b**	Cubes	0%	3%	49.834	28.500	0.088
		3%	0%	-49.834	28.500	0.088
	Boxes	0%	3%	173.720*	32.541	0.000
		3%	0%	-173.720*	32.541	0.000
**Carotenoids**	Cubes	0%	3%	6.664	10.318	0.522
		3%	0%	-6.664	10.318	0.522
	Boxes	0%	3%	66.170*	11.781	0.000
		3%	0%	-66.170*	11.781	0.000
**Sucrose x Support**						
**Dependent variable**	**Sucrose**	**I (Support)**	**J (Support)**	**Mean Difference (I-J)**	**Std. error**	**Significance**
**Chlorophyll a**	0%	Cubes	Boxes	-96.556*	42.689	0.029
		Boxes	Cubes	96.556*	42.689	0.029
	3%	Cubes	Boxes	108.430*	46.271	0.024
		Boxes	Cubes	-108.430*	46.271	0.024
**Chlorophyll b**	0%	Cubes	Boxes	-78.594*	29.332	0.010
		Boxes	Cubes	78.594*	29.332	0.010
	3%	Cubes	Boxes	45.293	31.793	0.161
		Boxes	Cubes	-45.293	31.793	0.161
**Carotenoids**	0%	Cubes	Boxes	-32.410*	10.620	0.004
		Boxes	Cubes	32.410*	10.620	0.004
	3%	Cubes	Boxes	27.096*	11.511	0.023
		Boxes	Cubes	-27.096*	11.511	0.023

Table S4. ANOVA tables corresponding to Figure 7, regarding the shoot height of willow plantlets cultured for 4 weeks in the phytotron and 6 weeks in the greenhouse in three types of soil.

A) ANOVA table corresponding to the first 4 weeks in the phytotron. Phy0: plant height after planting in pots and placing them in the phytotron. Phy2w, Phy4w, plant height after two (Phy2w) and four weeks (Phy4w) in the phytotron.

	**Source of Variation**	**Sum of squares**	**df**	**Mean square**	**F**	**Significance**
**Phy0w**	Between groups	0.400	1	0.400	0.474	0.499
	Within groups	18.599	22	0.845		
	Total	19.000	23			
**Phy2w**	Between groups	24.000	1	24.000	3.087	0.093
	Within groups	171.018	22	7.774		
	Total	195.018	23			
**Phy4w**	Between groups	264.007	1	264.007	16.432	0.001
	Within groups	353.458	22	16.066		
	Total	617.465	23	0.400		

B) Two-way ANOVA corresponding to the final stage of acclimation after 4 weeks in the phytotron and 6 weeks in the greenhouse in three types of soil. Factors are sucrose (the sucrose concentration during micropropagation) and the soil type (peat, forest soil and crop soil).

**Source of Variation**	**Sum of squares**	**df**	**Mean square**	**F**	**Significance**
**Sucrose**	29.704	1	29.704	1.638	0.217
**Soil type**	1269.966	2	634.983	35.026	0.000
**Sucrose x Soil type**	10.848	2	5.424	0.299	0.745

Table S5. Two-way ANOVA corresponding to basal diameter of willow plantlets cultured for 4 weeks in the phytotron and 6 weeks in the greenhouse in three types of soil. Factors are sucrose (the sucrose concentration during micropropagation) and the soil type (peat, forest soil and crop soil).

Source of Variation	Sum of squares	df	Mean square	F	Significance
**Sucrose**	0.002	1	0.002	0.006	0.941
**Soil type**	7.676	2	3.838	13.184	0.000
**Sucrose x Soil type**	0.841	2	0.420	1.444	0.262

Table S6. Two-way ANOVA corresponding to Figure 8, regarding the aerial and root biomass of willow plantlets cultured for 4 weeks in the phytotron and 6 weeks in the greenhouse in three types of soil. Factors are sucrose (the sucrose concentration during micropropagation) and the soil type (peat, forest soil and crop soil).

	Source of Variation	Sum of squares	df	Mean square	F	Significance
**Aerial biomass**	Sucrose	1.712	1	1.712	8.889	0.008
	Soil type	27.594	2	13.797	71.639	0.000
	Sucrose x Soil type	1.665	2	0.832	4.322	0.029
**Root biomass**	Sucrose	0.014	1	0.014	0.198	0.662
	Soil type	1.128	2	0.564	7.959	0.003
	Sucrose x Soil type	0.174	2	0.087	1.227	0.317

Pairwise comparisons for Aerial biomass after Bonferroni adjustment.

Sucrose x Soil type

Sucrose	I (Soil type)	J (Soil type)	Mean Difference (I-J)	Std. error	Significance
**0%**	Peat	Forest	2.835*	0.310	0.000
		Crop	2.540*	0.310	0.000
	Forest	Peat	-2.835*	0.310	0.000
		Crop	-0.295	0.310	1.000
	Crop	Peat	-2.540*	0.310	0.000
		Forest	0.295	0.310	1.000
**3%**	Peat	Forest	2.210*	0.310	0.000
		Crop	1.250*	0.310	0.002
	Forest	Peat	-2.210*	0.310	0.000
		Crop	-.960*	0.310	0.019
	Crop	Peat	-1.250*	0.310	0.002
		Forest	.960*	0.310	0.019

Soil type x Sucrose

Soil type	I (Sucrose)	J (Sucrose)	Mean Difference (I-J)	Std. error	Significance
**Peat**	0%	3%	1.172*	0.310	0.001
	3%	0%	-1.172*	0.310	0.001
**Forest**	0%	3%	0.547	0.310	0.095
	3%	0%	-0.547	0.310	0.095
**Crop**	0%	3%	-0.118	0.310	0.709
	3%	0%	0.118	0.310	0.709

Table S7. Two-way ANOVA corresponding to Figure 9, regarding the photosynthetic pigments of willow plantlets cultured for 4 weeks in the phytotron and 6 weeks in the greenhouse in three types of soil. Factors are sucrose (the sucrose concentration during micropropagation) and the soil type (peat, forest soil and crop soil).

	**Source of Variation**	**Sum of squares**	**df**	**Mean square**	**F**	**Significance**
**Chlorophyll a**	Sucrose	113506.260	1	113506.260	19.602	0.000
	Soil type	565328.581	2	282664.290	48.815	0.000
	Sucrose x Soil type	3633.271	2	1816.635	0.314	0.735
**Chlorophyll b**	Sucrose	43367.002	1	43367.002	21.015	0.000
	Soil type	185543.523	2	92771.762	44.956	0.000
	Sucrose x Soil type	1805.053	2	902.527	0.437	0.652
**Carotenoids**	Sucrose	7155.307	1	7155.307	17.751	0.001
	Soil type	65101.553	2	32550.776	80.752	0.000
	Sucrose x Soil type	138.681	2	69.340	0.172	0.843

Table S8. Two-way ANOVA corresponding to Table 2, regarding the enzymatic activity of the soils after being used for culturing willow plantlets during 6 weeks in the greenhouse. Factors are sucrose (the sucrose concentration during micropropagation) and the soil type (peat, forest soil and crop soil).

	**Source of Variation**	**Sum of squares**	**df**	**Mean square**	**F**	**Significance**
**Urease**	Sucrose	4.912	1	4.912	3.324	0.085
	Soil type	695.483	2	347.742	235.285	0.000
	Sucrose x Soil type	7.914	2	3.957	2.677	0.096
**Dehydrogenase**	Sucrose	0.017	1	0.017	11.603	0.003
	Soil type	0.534	2	0.267	185.683	0.000
	Sucrose x Soil type	0.017	2	0.009	5.944	0.052
**Acid phosphomonoesterase**	Sucrose	1.359	1	1.359	5.277	0.034
	Soil type	130.576	2	65.288	253.529	0.000
	Sucrose x Soil type	0.712	2	0.356	1.382	0.276
**ß-glucosidase**	Sucrose	0.051	1	0.051	2.477	0.133
	Soil type	54.271	2	27.135	1313.991	0.000
	Sucrose x Soil type	0.104	2	0.052	2.529	0.108
**Invertase**	Sucrose	0.397	1	0.397	2.092	0.165
	Soil type	156.767	2	78.384	413.208	0.000
	Sucrose x Soil type	0.489	2	0.244	1.288	0.300
**Arylsulfatase**	Sucrose	0.000	1	0.000	0.000	1.000
	Soil type	0.466	2	0.233	235.680	0.000
	Sucrose x Soil type	0.000	2	0.000	0.233	0.795
